# Dietary and Lifestyle Factors Modulate the Activity of the Endogenous Antioxidant System in Patients with Age-Related Macular Degeneration: Correlations with Disease Severity

**DOI:** 10.3390/antiox9100954

**Published:** 2020-10-05

**Authors:** Zofia Ulańczyk, Aleksandra Grabowicz, Elżbieta Cecerska-Heryć, Daria Śleboda-Taront, Elżbieta Krytkowska, Katarzyna Mozolewska-Piotrowska, Krzysztof Safranow, Miłosz Piotr Kawa, Barbara Dołęgowska, Anna Machalińska

**Affiliations:** 1 Department of General Pathology, Pomeranian Medical University, 70-111 Szczecin, Poland; zofia.litwinska@pum.edu.pl (Z.U.); kawamilosz@gmail.com (M.P.K.); 2 First Department of Ophthalmology, Pomeranian Medical University, 70-111 Szczecin, Poland; aleksandra.grabowicz@pum.edu.pl (A.G.); oko1@pum.edu.pl (E.K.); kmp@pum.edu.pl (K.M.-P.); 3 Department of Laboratory Medicine, Pomeranian Medical University, 70-111 Szczecin, Poland; cecerskaela@wp.pl (E.C.-H.); daria.sleboda@pum.edu.pl (D.Ś.-T.); barbara.dolegowska@pum.edu.pl (B.D.); 4 Department of Biochemistry and Medical Chemistry, Pomeranian Medical University, 70-111 Szczecin, Poland; chrissaf@mp.pl

**Keywords:** age-related macular degeneration (AMD), antioxidants, diet

## Abstract

Age-related macular degeneration (AMD) is a common cause of blindness in the elderly population, but the pathogenesis of this disease remains largely unknown. Since oxidative stress is suggested to play a major role in AMD, we aimed to assess the activity levels of components of the antioxidant system in patients with AMD. We also investigated whether lifestyle and dietary factors modulate the activity of these endogenous antioxidants and clinical parameters of disease severity. We recruited 330 patients with AMD (39 with early, 100 with intermediate and 191 with late form of AMD) and 121 controls in this study. At enrolment, patients’ dietary habits and physical activity were assessed, and each study participant underwent a thorough ophthalmologic examination. The activity of several components of the antioxidant system were measured in red blood cells and platelets using both kinetic and spectrophotometric methods. Patients with AMD consumed much lower levels of fatty fish and eggs than the control group (*p* = 0.008 and *p* = 0.04, respectively). In the nAMD group, visual acuity (VA) correlated positively with green vegetable consumption (Rs = +0.24, *p* = 0.004) and omega-3-rich oil intake (Rs = +0.17, *p* = 0.03). In the AMD group, the total physical activity MET score correlated positively with VA (Rs = +0.17, *p* = 0.003) and correlated negatively with the severity of AMD (Rs = −0.14, *p* = 0.01). A multivariate analysis of patients and controls adjusted for age, sex, and smoking status (pack-years) revealed that AMD was an independent variable associated with a lower RBC catalase (β = −0.37, *p* < 0.001) and higher PLT catalase (β = +0.25, *p* < 0.001), RBC GPx (β = +0.26, *p* < 0.001), PLT GPx (β = +0.16, *p* = 0.001), RBC R-GSSG (β = +0.13, *p* = 0.009), PLT R-GSSG (β = +0.12, *p* = 0.02) and RBC GSH transferase (β = +0.23, *p* < 0.001) activity. The activities of components of the antioxidant system were associated with disease severity and depended on dietary habits. The observed substantial increase in the activity of many critical endogenous antioxidants in patients with AMD further indicates that the required equilibrium in the antioxidant system is disturbed throughout the course of the disease. Our findings explicitly show that a diet rich in green vegetables, fish and omega-3-rich oils, supplemented by physical exercise, is beneficial for patients with AMD, as it might delay disease progression and help retain better visual function.

## 1. Introduction

Age-related macular degeneration (AMD) is an incurable ocular condition of the outer retina that affects approximately 8–10% of the elderly population worldwide [1]. AMD is characterised by a progressive visual impairment and remains the leading cause of visual disability in developed countries [2]. Because of the increasing life expectancy, the number of individuals with AMD is estimated to reach 288 million in 2040 [3,4]. This increasing prevalence of AMD represents a major financial challenge for healthcare systems and is expected to exert increasing socio-economic effects [5].

As the pathogenesis of AMD is still poorly understood, the treatment options remain limited and are available only for the advanced, neovascular form of AMD (nAMD) [6]. The antiangiogenic treatment targets the main pathophysiological feature of this subtype of AMD: the formation of new, largely malformed vessels (choroidal neovascularization, CNV) [7]. At present, no proven treatment is available for the earlier stages of AMD nor for GA, which are characterised by the accumulation of drusen and retinal atrophy [8]. Therefore, research has focused on the prevention and/or slowing the progression of AMD to its late stages by manipulating modifiable risk factors, among which nutrition and dietary habits are listed as the most important risk factors [9]. Based on research evidence, changes in a patient’s dietary habits and the addition of supplements represent a simple and cost-effective method for modifying the risk of developing and progression of AMD [10]. Several observational and experimental studies have been conducted in humans to investigate associations between dietary antioxidants, the consumption of certain foods and AMD [11], including Age Related Eye Disease Study 1 (AREDS1) [12]. Two of the most notable studies, AREDS1 and most recently AREDS2, have contributed to the supplementation strategy currently used in clinical practice [13]. These studies have noted the effects of dietary factors such as omega-3s, carotenoids, lutein/zeaxanthin, vitamins A and D on eye health and suggested that they might affect the course of AMD. These findings resulted in the development of recommendations and clinical practice guidelines that have been used as a decision-making tool in clinical settings [14]. In general, the most cost-effective and seemingly achievable strategy for the prevention of progression of AMD to its later stages appears to be a general healthy lifestyle that is achieved by a healthy diet and exercise [15,16].

The pathogenesis of AMD has been attributed to both modifiable and unmodifiable factors, including age, genetics and active smoking [17]. The most well-known genetic factors associated with an increased risk of AMD are polymorphisms in the *CFH* Y402H (complement factor H) [18,19] and *ARMS2* (age-related maculopathy susceptibility 2) genes [20]. Recently, our team identified another variant associated with an increased risk for AMD that is located in the peripherin-2 (*PRPH2*) gene, which encodes a photoreceptor-specific protein vital for rod and cone cell formation and stability [21]. The detrimental effect of the major modifiable risk factor for AMD, cigarette smoking, which increases the risk of AMD 2–4 times [22,23,24], has been attributed to the induction of angiogenesis, impairments in choroidal circulation, activation of the immune system and generation of oxidative damage [22,25,26]. In fact, risk factors other than smoking that contribute to AMD development, such as light exposure, diet, and vitamin D levels, among others, also exert well-documented effects on oxidative stress, which corresponds to cellular damage caused by reactive oxygen species (ROS) [27].

At the molecular level, the retinal environment is particularly susceptible to oxidative stress, as it is constantly exposed to light and is characterised by increased oxygen consumption and a high proportion of polyunsaturated fatty acids [27,28]. ROS directly damage DNA (particularly in mitochondria) and lipids in the photoreceptors, leading to the deterioration of the retinal pigment epithelium (RPE) [29]. These oxidatively damaged molecules then accumulate in the macular area and become a continuous source of chronic oxidative stress [30]. The retina protects itself from oxidative damage by producing a considerable number of antioxidants in the photoreceptor and RPE cells, including the enzymes superoxide dismutase (SOD), glutathione peroxidase (GPx), catalase (CAT), glutathione transferase (GST) and glutathione reductase (R-GSSG) and nonenzymatic antioxidants glutathione (GSH), carotenoids, uric acid, albumin and many others [31]. Disturbances in the tight balance of antioxidant system might contribute to AMD pathogenesis [32].

In the present study, we aimed to assess the activity levels of several components of the antioxidant system in patients with AMD and controls and to explore the role of dietary habits in AMD development. In particular, we wanted to investigate whether lifestyle factors modulate the concentrations of these endogenous antioxidants and clinical parameters of disease severity. We also focused on possible associations of antioxidant activity with genetic risk factors for AMD.

## 2. Materials and Methods

### 2.1. Study Groups and Initial Management

For this study we recruited 330 patients with AMD (39 with early, 100 with intermediate and 191 with late form of AMD) from the outpatient population of the First Department of Ophthalmology of Pomeranian Medical University in Szczecin, Poland (ethical code is KB-0012/141/13). For the control group, we enrolled 121 age-matched participants with no symptoms or signs of macular degeneration (absence of drusen, neovascularization or pigmentary abnormalities). We excluded patients with significant chronic systemic conditions (diabetes mellitus, renal failure, collagen/neoplastic disease, hepatic dysfunction, etc.) or ongoing retinal disease except AMD (in AMD groups), i.e., glaucoma or intraocular inflammatory diseases from the participation in the study. A consent form was signed by all patients before enrolment in the trial, in accordance with the tenets of the Declaration of Helsinki.

Demographic characteristics (age, gender, time of symptom onset, time to presentation) and the following vascular risk factors present at the time of the enrolment were recorded: hypertension, hyperlipidaemia, diabetes mellitus, atrial fibrillation, ischaemic heart disease, cardiomyopathy, prior cerebrovascular events or renal artery stenosis (atherosclerosis). The following anthropometric and nutritional parameters were also assessed in all patients: waist circumference [cm], waist/hip ratio (WHR), and body mass index (BMI) [weight (kg)/height (m)^2^]. Cumulative pack-years of smoking were determined from the recorded average number of smoked cigarettes per day and smoking years. The actual blood pressure (BP) was determined in all subjects prior to the ophthalmic examination.

### 2.2. Dietary Habits and Physical Activity Assessment

At the enrolment, each patient completed Food Frequency Questionnaire (FFQ) and International Physical Activity Questionnaire (IPAQ) with the help of the member of the research team.

We modified a quantitative Food Frequency Questionnaire (FFQ) to assess the intake of the following food groups rich in nutrients considered important in the AMD aetiology and oxidative processes to evaluate the participants’ dietary habits: fatty fish, eggs, green vegetables, fruit and fruit juice, omega-3-rich oils, simple and complex carbohydrates, as recommended previously [11,13,33,34]. Three different frequencies in terms of portions per week were available for selection for each food type and alcoholic drink as follows: fatty fish: <1, 2–4, and >4; eggs: <1, 2–4, and >4; green vegetables: <2, 2–7, and >7; fruit and fruit juice: <2, 2–7, and >7; omega-3-rich oils: <2, 2–7, and >7; simple carbohydrates: <2, 2–7, and >7; complex carbohydrates: <2, 2–7, and >7; beer: 0, ≤1, and 2–7; wine: 0, <2, and 2–7; and vodka: 0, ≤1, and 2–3.

Each participant completed the International Physical Activity Questionnaire (IPAQ) with the assistance of the member of the research team due to participants’ vision impairment, which comprises 7 questions regarding all types of physical activity associated with daily life, work and leisure performed in the last seven days. The duration of each activity included in the final data was 10 min or longer with no interruptions at any moment. The physical activity score was presented in MET-min per week units and was calculated by multiplying a factor specific for each activity by several days spent performing the activity and time in min spent on the activity per day. Weekly activity was measured by adding scores of each of the activities.

### 2.3. Ophthalmologic Examination

The patients were examined by an ophthalmologist with a comprehensive ophthalmologic evaluation, including best-corrected visual acuity using the Early Treatment Diabetic Retinopathy Study (ETDRS) chart, intraocular pressure measurement, fundus photography, autofluorescence imaging, spectral-domain OCT, and fluorescein or indocyanine green angiography (Spectralis, Heidelberg Engineering, Carlsbad, CA, USA). The severity of AMD was classified according to Ferris et al. [35]: patients with medium drusen (63–125 m) and without pigmentary abnormalities were classified as early AMD group, patients with large drusen or with pigmentary abnormalities associated with at least medium drusen were classified as intermediate AMD group, and patients with lesions associated with neovascular AMD or geographic atrophy were classified to have late AMD. The examinations were carried out in a blinded manner.

### 2.4. Blood Sample Collection, RBC and PLT Preparation

Peripheral venous blood (approx. 7.5 mL) was collected from the AMD group and controls into two types of tubes containing ethylenediaminetetraacetic acid (EDTA) or sodium citrate as an anticoagulant. The blood sample in the EDTA tube was centrifuged at 3000× *g* for 10 min to separate the plasma and buffy coat from red blood cells (RBCs). The plasma and buffy coat were removed from RBCs and 3 mL of deionised water were added to induce haemolysis. The sample was then centrifuged at 13,500× *g* for 5 min to separate the haemolysate from the pellet of red blood cell membranes. Haemoglobin levels were assayed using Drabkin’s method. All results obtained for the activity of antioxidant enzymes were calculated per 1 g of haemoglobin in RBCs.

Platelets were obtained from venous blood collected in a tube containing 109 mM sodium citrate (3.2%, 9:1; *v*/*v*). Blood was centrifuged (10 min; 20 °C; 10,000 rpm) to obtain platelet-rich plasma (PRP), which was transferred to a new tube and centrifuged again (10 min; 20 °C; 3824 rpm). The resulting platelet-poor plasma (PPP) was placed in a fresh tube and stored at −80 °C until further analysis. The platelet pellet was washed twice with Tyrode’s solution (pH 7.4), suspended in 1 mL of Tyrode’s solution and the number of platelets was determined using spectrophotometry. The suspension was stored at −80 °C until further analysis. The platelet suspension was thawed (37 °C) and frozen (−80 °C) twice, and the obtained platelet lysate was centrifuged (10 min; 4 °C; 3824 rpm). All results obtained for the activity of antioxidant enzymes were calculated per 1 g of platelet lysate protein. Protein levels were assayed using the Lowry protein assay.

We used automated methods and commercially available assays to measure fasting glucose and lipid levels (including triglycerides, total cholesterol and high (HDL) and low-density (LDL) lipoproteins) in all patients with AMD and controls.

### 2.5. The Activity of Antioxidant Enzymes

A spectrophotometric method was used to establish the concentrations of reduced glutathione (GSH). The activities of superoxide dismutase (SOD), catalase (CAT), glutathione peroxidase (GPx), glutathione transferase (GST), glutathione reductase (R-GSSG) in red blood cells (RBCs) and platelets (PLT) were obtained using kinetic methods. The measurements in RBCs and PLT were performed using a UV/VIS Lambda 650 spectrophotometer (Perkin-Elmer, Waltham, MA, USA) and similar protocols (specified below). The extracellular haemoglobin concentration in plasma samples was determined using a spectrophotometric method [36,37] with the same spectrophotometer.

#### 2.5.1. GSH Concentration

The (haemo)lysate was diluted, mixed with a precipitation solution (1.67 g of metaphosphoric acid, 0.2 g of EDTA-Na_2_, 30 g of NaCl and 100 mL of H_2_O; Sigma-Aldrich, St. Louis, MO, USA), incubated (5 min, 4 °C) and centrifuged (550× *g* for 10 min). The supernatant was diluted with phosphoric buffer (pH 7.9), DTNB (5,5’-dithiobis-(2-nitrobenzoic acid), Sigma-Aldrich, St. Louis, MO, USA) was added, and then the mixture was incubated for 15 min at 25 °C. The detection wavelength was λ 412 nm. The GSH concentration was calculated using the molar absorption coefficient (e = 13,600 M^−1^ cm^−1^).

#### 2.5.2. SOD Activity

In a test tube, a mixture of (haemo)lysate, chloroform:ethanol (3:5; *v*/*v*) solution, and distilled water was combined. The mixtures were subsequently vortexed and centrifuged (5 min; 4 °C; 3824× *g*). The study material (Na_2_CO_3_/NaHCO_3_ buffer, SOD extract and adrenaline solution; Sigma-Aldrich, St. Louis, MO, USA) was incubated for 3 min at 37 °C. The absorbance of the study material was recorded in 5 min at a wavelength of 320 nm (in 30 °C).

#### 2.5.3. CAT Activity

The (haemo)lysate was diluted 500-fold (100-fold for PLT) with 50 mM phosphoric buffer. Absorbance measurements of the study sample ((haemo)lysate and 30 mM H_2_O_2_ solution; Sigma-Aldrich, St. Louis, MO, USA) were performed within 30 s at a wavelength of 240 nm (at 30 °C). CAT activity was determined from a calibration curve, which was obtained as a result of assays of several solutions with a known catalase activity pattern (Oxis Research, Portland, OR, USA).

#### 2.5.4. GPx Activity

A reaction mixture was prepared (phosphoric buffer, glutathione reductase, GSH, NADPH + H^+^, and (haemo)lysate with transforming reagent incubated for 5 min at room temperature beforehand; transforming reagent used only for RBC) and incubated for 10 min at 37 °C. After the incubation, the reaction was initiated by adding tert-butyl hydroxide (or hydrogen peroxide), and the decrease in the absorption at λ 340 nm was measured. The amount of enzyme that oxidised 1 µmol of GSH (0.5 µmol NADPH + H) in one minute was defined as a unit of enzyme activity.

#### 2.5.5. GST Activity

A reaction mixture was combined (phosphoric buffer, GSH, CDNB (1-chloro-2,4-dinitrobenzene), and (haemo)lysate), and the increase in absorbance at λ 340 nm was measured. Glutathione transferase activity was determined using molar absorption coefficient of the synthesised conjugate (e = 9600 M^−1^ cm^−1^).

#### 2.5.6. R-GSSG Activity

The (haemo)lysate was diluted, mixed with 1 mL of a diluted RI working reagent (900µL EDTA and 100µL RI; RI: NADPH^+^ + H^+^ diluted in 0.01 M NaOH, Sigma Aldrich, St. Louis, MO, USA) and incubated (5 min, 30 °C). Then, RII reagent was added (GSSG (glutathione disulphide) diluted in EDTA) and extinction was measured at λ 340 nm over 3 min at 30 °C.

#### 2.5.7. Statistical Analysis

The quantitative parameters measured in both eyes were averaged before further analysis. The distributions of all the analysed variables related to components of antioxidant system were right-skewed and significantly different from normal distribution (*p* < 0.05, Shapiro-Wilk test). Therefore, the nonparametric Kruskal-Wallis and Mann-Whitney tests were used to compare quantitative values between groups, whereas Spearman’s rank correlation coefficient (Rs) was calculated to measure the strength of associations between these values. We used Fisher’s exact test to compare qualitative variables between groups. A multivariate analysis of AMD as an independent variable was performed using a general linear model (GLM) adjusted for age, sex and smoking status (pack-years) by inclusion of the confounding factors as independent variables, with logarithmic transformation applied to the dependent variables to normalize their distributions. Standardised regression coefficients (β) were calculated to measure the strength of associations between independent and dependent variables. The interpretation of β and Rs coefficients is similar: values +1 and −1 indicate perfect positive and negative association, respectively, while 0 indicates complete lack of association. Quantitative variables were presented as mean ± standard deviation. *p* < 0.05 was considered statistically significant without correction for multiple testing other than included in the applied test itself (number of compared groups in Kruskal-Wallis test, number of variables in GLM). Statistica 13 software (Dell Inc., Round Rock, TX, USA) was used for statistical analyses.

## 3. Results

### 3.1. Characteristics of the Study Subjects

We enrolled 330 patients with AMD and 121 healthy controls in this study. The clinical characteristics of the studied groups are presented in Table 1. The AMD and control groups did not differ in age and well-known atherosclerotic risk factors, including serum lipid and glucose levels. Statistically significant differences in physical activity were not observed between the groups. On the other hand, the number of past smokers and the number of pack-years of smoking were considerably higher in the AMD group than in controls (*p* < 0.001). Importantly, a strong positive correlation between the number of pack-years of smoking and disease severity was identified (Rs = +0.23, *p* < 0.001), corroborating the well-documented relation between smoking and AMD. The positive correlation between the patient’s age and clinical classification of AMD (Rs = +0.19, *p* < 0.001) indicates that age is one of the main factors affecting disease severity.

On the other hand, the HDL level correlated negatively with disease severity (Rs = −0.15, *p* = 0.007), suggesting its protective effect on AMD progression. Another negative correlation was observed between the education level and disease severity (Rs = −0.14, *p* = 0.01). Thus, a higher educational attainment might be associated with better health awareness and subsequently reduce AMD progression.

### 3.2. Analysis of Lifestyle Habits

Since diet is considered one of the potentially modifiable risk factors for AMD, we aimed to compare the dietary habits of patients with AMD and healthy controls (Table 2) and to analyse whether dietary habits differed between the early, intermediate and late AMD groups (Table 3).

Patients with AMD consumed much less fatty fish than the control group (*p* = 0.008). Similar findings were observed for egg consumption (*p* = 0.04). In contrast, the AMD group recorded higher consumption of fruits and fruit juices than the controls (*p* = 0.01). We did not observe significant differences in the consumption of green vegetables, omega-3 rich oils, and simple and complex carbohydrates between groups (*p* = 0.39, *p* = 0.66, *p* = 0.18, and *p* = 0.26, respectively). Accordingly, no differences in alcohol intake were observed between groups. Interestingly, we observed a significantly higher consumption of fatty fish in patients with intermediate AMD than in patients with the late form of the disease (*p* = 0.01). Significant differences in egg, green vegetable, fruit and fruit juice, omega-3-rich oil, simple and complex carbohydrate consumption and alcohol intake were not observed between the early, intermediate and late AMD groups.

We aimed to determine whether physical activity and the consumption of specific food groups or alcohol were associated with disease severity to better assess the roles of lifestyle and dietary habits in the progression of AMD. In the AMD group, we observed strong positive correlations between VA and the total physical activity MET score (Rs = +0.17, *p* = 0.003), but these correlations were not observed in controls. Positive correlations between VA and physical activity intensity were clearly detectable in the nAMD group, including the intense physical activity MET score (Rs = +0.17; *p* = 0.04), average physical activity MET score (Rs = +0.21; *p* = 0.01) and total physical activity MET score (Rs = +0.22, *p* = 0.006). Thus, patients with AMD who were more physically active displayed better visual function. Accordingly, the total physical activity MET score negatively correlated with the severity of AMD (Rs = −0.14, *p* = 0.01). Similarly, time (in min) spent sitting in the last 7 days was associated with more advanced stages of AMD (Rs = +0.20, *p* = 0.0005). Based on these findings, sedentary behaviour facilitates the progression of AMD.

Regarding dietary factors, VA correlated positively with green vegetable consumption (Rs = +0.24, *p* = 0.004) and omega-3-rich oil intake (Rs = +0.17, *p* = 0.03) in the nAMD group. Therefore, the consumption of these food products might preserve visual function in patients with nAMD, although no differences in the consumption of those products were observed between the AMD and control groups. Accordingly, fatty fish consumption correlated positively with the retinal volume in the AMD group (Rs = +0.23, *p* = 0.003). We observed negative correlations between the drusen size and consumption of green vegetables (Rs = −0.19, *p* = 0.02), fruit and fruit juice (Rs = −0.28, *p* = 0.0004), omega-3-rich oils (Rs = −0.24, *p* = 0.002) and complex carbohydrates (Rs = −0.21, *p* = 0.01) in the AMD group. These correlations were not observed in the control group. Thus, the consumption of these products might exert beneficial effects on the disease course and reduce AMD progression.

### 3.3. Components of the Antioxidant System

**Figure 1:**
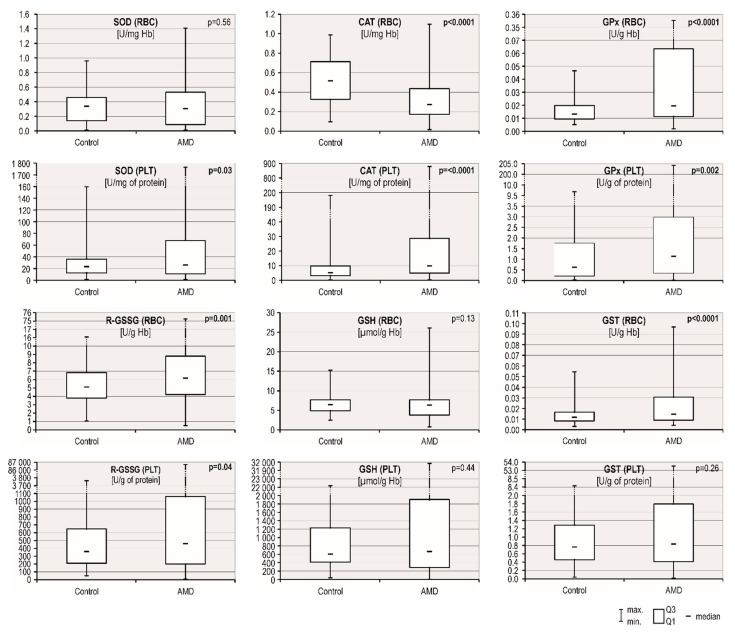
Comparison of the antioxidant system component levels in patients with AMD and controls. *p*-values < 0.05, which were considered statistically significant, are shown in bold.

Excess oxidative stress coupled with overwhelmed antioxidant defence systems are thought to be the important contributors to the complex pathophysiology of AMD. We chose 6 factors to analyse the efficiency of the antioxidant system in patients with AMD and controls: the activities of five enzymes (SOD, CAT, GPx, R-GSSG and GSH transferase) and concentrations of reduced glutathione (GSH) in red blood cells (RBCs) and platelets (PLT). The AMD group presented higher values for 7 of the 12 tested factors compared with the control group (Table 4, Figure 1): GPx, R-GSSG, GSH transferase in RBCs and SOD, catalase, GPx and R-GSSG in PLT. A significant downregulation in catalase activity levels was observed in RBCs from patients with AMD (0.33 ± 0.21 U/mg Hb) compared to controls (0.53 ± 0.24; *p* < 0.0001). A multivariate analysis of patients and controls adjusted for age, sex, and smoking status (pack-years) revealed that AMD was an independent variable associated with a lower RBC catalase (β = −0.37, *p* < 0.001) and higher PLT catalase (β = +0.25, *p* < 0.001), RBC GPx (β = +0.26, *p* < 0.001), PLT GPx (β = +0.16, *p* = 0.001), RBC R-GSSG (β = +0.13, *p* = 0.009), PLT R-GSSG (β = +0.12, *p* = 0.02) and RBC GSH transferase (β = +0.23, *p* < 0.001) activity levels.

An analysis stratified by AMD severity revealed that the early AMD group presented higher GSH (RBC) concentration and lower R-GSSG (PLT) activity than the late AMD group (*p* = 0.03 and *p* = 0.04, respectively) (Table 5). However, we should be rather careful in interpreting the results of the Mann–Whitney test, since the Kruskal-Wallis test showed no statistically significant differences between the early, intermediate and late AMD groups.

Next, we investigated the associations between ophthalmic parameters and concentrations of the analysed antioxidants to more specifically evaluate the functions of antioxidants in the development of AMD. In the AMD group, RBC catalase activity and GSH concentrations negatively correlated with the disease severity (Rs = −0.11, *p* = 0.04; R = −0.11, *p* = 0.05, respectively). This relationship corresponds to lower RBC catalase activity in the AMD group than in controls. Similarly, we observed weak positive correlations between the clinical classification of AMD and RBC GPx (Rs = +0.10, *p* = 0.07), PLT catalase (Rs = +0.10, *p* = 0.08) and R-GSSG PLT (Rs = +0.10, *p* = 0.08) activities that corresponded to higher activities of these enzymes in RBCs and PLT from patients with AMD. Accordingly, the drusen size in patients with AMD correlated positively with SOD, GPx and GSH transferase activities in RBCs (Rs = 0.31, *p* < 0.001; Rs = 0.16, *p* = 0.003; and Rs = 0.18, *p* < 0.001, respectively) and negatively correlated with both RBC activity and PLT GSH concentration (Rs = −0.27, *p* < 0.001; and Rs = −0.24, *p* < 0.001, respectively). This association was similar to the higher RBC GPx and GSH transferase activity in the AMD group than in controls. Based on these results, the antioxidant system might be a major contributor to the clinical course of AMD.

### 3.4. Correlations between the Antioxidant System and Lifestyle Factors

Different nutritional factors are proposed to modulate the antioxidant potential of various cells [38,39]. We evaluated the possible associations between the activity levels of components of the antioxidant system with physical activity and diet to assess whether the activities of the investigated antioxidant enzymes in RBCs and PLT were associated with lifestyle factors and whether these correlations were specific for patients with AMD. Overall, GPx, R-GSSG and GSH transferase activities in RBCs from the AMD group correlated negatively with egg consumption (Rs = −0.22, *p* < 0.001; Rs = −0.17, *p* = 0.003; and Rs = −0.13, *p* = 0.03, respectively), whereas the RBC catalase activity was positively correlated with the amount of egg consumption (Rs = +0.11, *p* = 0.05). Similar correlations were not observed in controls. These relationships clearly correspond to higher GPx, R-GSSG and GSH transferase activities and lower catalase activity in the AMD group, as well as lower egg consumption, than in controls. Similarly, in the AMD group, positive correlations between RBC catalase activity and fatty fish consumption (Rs = +0.18, *p* = 0.001) paralleled the lower consumption of this dietary product and lower catalase activity in patients with AMD than in controls. Accordingly, we observed positive correlations between fruit and fruit juice consumption with two PLT enzymes: SOD and GPx (Rs = +0.12, *p* = 0.03 and Rs = +0.14, *p* = 0.02, respectively). This finding confirms the aforementioned observation, since the activities of both of these enzymes and fruit and fruit juice consumption were higher in the AMD group than in the control group. The identified relationships indicate the possible effect of dietary habits on antioxidant activity in patients with AMD.

In terms of physical activity, R-GSSG activity levels in RBCs correlated negatively with the total physical activity MET score (Rs = −0.17, *p* = 0.04) in patients with nAMD, but this correlation was not observed in controls.

### 3.5. Genotypes and Components of the Antioxidant System

We also explored the relationship between antioxidant activity and genetic risk factors for AMD to further elucidate the interactions between various risk factors for AMD and their contributions to disease pathogenesis. For this purpose, we investigated associations between the six selected antioxidants and polymorphisms in genes previously associated with AMD: *CFH* Y402H, *ARMS* A69S and a single nucleotide variant that our team recently linked to a higher AMD risk, *PRPH2* c.582-67T > A (rs3818086) (paper in press). However, when the correction for multiple testing was applied, we did not identify any statistically significant relationships between components of the antioxidant system and the genotypes of these genes.

## 4. Discussion

AMD remains the major cause of visual impairment among the elderly population, significantly reducing the quality of life of affected individuals [40]. Several genetic and environmental factors, including the *CFH* Y402H polymorphism, age and cigarette smoking, have been identified as contributing to the complex landscape of AMD, although the exact pathogenesis of the disease remains unclear [41,42]. At the molecular level, various risk factors for AMD share a common denominator, oxidative stress, which is thought to be the main component of AMD pathology [27,43]. In fact, manipulations of dietary and lifestyle habits, which are thought to contribute to the tight balance of the endogenous antioxidant system, might be beneficial in preventing and/or slowing the progression of AMD [11]. Thus, in the present study, we aimed to investigate the role of antioxidant components in AMD and to assess whether dietary and lifestyle factors modulate the levels of those endogenous antioxidants and clinical parameters of disease severity. We also assessed possible relationships between antioxidant activity and genetic risk factors for AMD.

First, we assessed the systemic levels of components of the antioxidant system in peripheral blood and found that the activity of the majority of tested substances were significantly increased in patients with AMD (GPx, R-GSSG, and GSH transferase levels in RBCs and SOD, CAT, GPx, and R-GSSG levels in PLT), whereas only the CAT activity in RBC was evidently reduced in patients with AMD compared with controls. Our observation of increased GPx activity is in contrast to the results reported by Mrowicka et al. [44] and Plestina-Borjan et al. [31], where significantly lower GPx (RBC) activity was observed in AMD patients in comparison with controls. On the other hand, in the large POLA study of a cohort of 2584 participants, the increased levels of plasma GPx concentration, which catalyses H_2_O_2_ degradation by GSH, correlated with a nine-fold increase in the prevalence of late AMD [45]. GPx not only protects RPE cells in models of oxidative damage-induced retinal degeneration but is also required for the maturation of photoreceptor cells [46]. As proposed by Tokarz et al., the increased activity of GPx in patients with AMD reflects the activity of RPE cells, which attempt to dispose of overwhelming amount of H_2_O_2_ formed during the disease course [47]. R-GSSG interacts with GPx to regulate the GSH concentration, as it converts glutathione disulfide (GSSG) to GSH [48]. In contrast to our results, the R-GSSG concentration and activity have been reported to be rather low in patients with AMD and was associated with a decrease in GSH levels [49,50], the product of enzymatic reaction catalysed by R-GSSG. In our study, we did not observe a significant association between R-GSSG activity and GSH concentration in any of the tested groups; thus, the increase in R-GSSG activity in patients with AMD might be an analogous indicator of increased antioxidant activity, similarly to GPx or increased GSH transferase activity. Interestingly, conflicting results on the involvement of *GSTM1* and *GSTM5* (glutathione s-transferase mu 1/5) polymorphisms and gene expression in AMD pathology exist [51,52], once again suggesting that the necessary balance of the antioxidant system is achieved through the proper activity of several enzymes, and not a single enzyme. The tight cooperation of endogenous antioxidants is reflected in SOD activity, which functions together with GPx and CAT to convert H_2_O_2_ to nontoxic products and by that protect the photoreceptors and RPE from oxidative damage [31,49]. SOD activity decreases in the RPE periphery with ageing and at the same time its immunoreactivity increases [53]. However, in the aforementioned POLA study, a high level of erythrocyte SOD activity was not associated with AMD [45]. This finding is consistent with our results, although we also observed a higher activity of SOD in PLT from the AMD group. On the contrary, Venza et al. reported lower SOD (both in plasma and RBC) activity in AMD patients compared to controls [54]. Interestingly, in vitro studies have shown a reduction in SOD activity in response to oxidative stress when ARPE-19 cells were treated with acrolein, a powerful initiator of oxidative stress and mitochondrial dysfunction [55], whereas the upregulation of *SOD1/2* expression resulted in oxidative damage in RPE cells [56]. In fact, excess SOD (in relation to the activities of GPx and CAT) may cause damage [57], which further suggests the need for a tight balance of the antioxidant system. Similar to SOD, CAT function decreases in the macular and peripheral RPE with ageing [58], but in contrast to SOD, CAT immunoreactivity is reduced in RPE cells in the eyes of patients with and without AMD [47,59]. Our finding of reduced CAT activity in patients with AMD is consistent with previous reports [44]. As proposed by Tate et al., treatment with ROS-generating compounds induces CAT expression in RPE cells, which protects against H_2_O_2_, even in the adjacent RPE cells without upregulated CAT expression. Overall, our findings of increased activity of several antioxidants in the AMD group suggest an enhanced response to oxidative damage that might contribute to AMD pathogenesis by disrupting the tight balance of the antioxidant system [32].

Dietary antioxidants aid the endogenous antioxidant defence system in protecting against oxidative damage and enhanced ROS production and consequently, prevent or slow related disorders, including AMD [60]. Several major clinical trials, in particular the Age-Related Eye Disease Study (AREDS) and AREDS2, have shown that nutrients with antioxidant properties, namely, lutein, zeaxanthin, polyunsaturated omega-3 fatty acids (PUFAs), zinc, vitamins C and E, delay the progression of advanced AMD in persons with intermediate AMD [13,61]. In our study, patients with AMD consumed much lower levels of fatty fish and eggs than controls, whereas greater consumption of green vegetables and omega-3-rich oils was correlated with favourable clinical outcomes (better visual acuity and a smaller drusen size in patients with AMD). These observations are consistent with previous studies, as patients with AMD are generally encouraged to increase their intake of green vegetables, eggs and fish [9,14,33]. Both green vegetables and eggs are rich sources of lutein and zeaxanthin, potent anti-inflammatory and antioxidant factors that exert beneficial effects on slowing the progression of AMD [15]. The antioxidant potential of these macular carotenoid pigments combined with their ability to filtrate blue-light may serve not only to protect the ocular tissue from oxidative damage, but also to improve visual acuity [62]. Our study further supports this notion, as we found a positive correlation between VA and consumption of green leafy vegetables, which are a well-known source of these potent macular carotenoid pigments [63]. According to Gopinath et al., eggs also contain large quantities of selenium, which directly protects cells from oxidative damage [64]. Additionally, eggs, similar to fatty fish, are a good source of omega-3 PUFAs, which may minimize retinal inflammation, oxidation, and degeneration [10,64,65]. Indeed, the Blue Mountains Eye Study (BMES) of a large Australian cohort has shown that a greater consumption of fish and omega-3s may slow AMD progression [66]. Interestingly, we did not observe any correlations between alcohol consumption and the clinical parameters in our patients, although at the molecular level, the toxicity of alcohol is associated with lipid peroxidation and oxidative stress and potentially represents another causative factor for AMD [67]. This finding is consistent with a study by Knudtson et al. [68], although some recent reports suggest a modest association between alcohol consumption and an increased AMD risk [69].

The preventive strategies incorporating a modification of the diet represent an attractive approach to slow AMD progression, but some debate exists in the literature regarding whether physical exercise is also recommended to protect against AMD [70]. Our study provides a clear indication that a sedentary lifestyle worsens the AMD course, as more physically active patients presented better visual acuity. Several previous studies have associated physical activity with a lower risk of AMD [71,72], but reports have also described the lack of significant relationships between exercise and AMD risk [73]. As shown in a recent study by Gopinath et al., the most physically active patients aged at least 75 years are 79% less likely to develop late AMD in 15 years [70]. However, when other confounding factors were considered, no significant association was observed in this group. Overall, the systemic benefits of physical activity (e.g., protective effects on obesity, diabetes, inflammation, etc.) make it a vital part of a healthy lifestyle [70,74], which should be recommended to patients with AMD, along with refraining from cigarette smoking and consuming a diet rich in vegetables, fish and eggs.

It is worth noting; however, that the correlations found in the present study do not necessarily indicate causation. Thus, further studies on larger cohorts are needed to provide more straightforward evidence of various dietary and lifestyle factors affecting AMD course.

## 5. Conclusions

In our study, we observed significant increases in the levels of several crucial endogenous antioxidants in blood samples from patients with AMD, which further suggests that the necessary balance of the antioxidant system is disrupted during the disease course. Since dietary habits potentially modulate AMD progression by contributing to the antioxidant status, we also investigated food intake in our cohort and identified several significant correlations. Based on our results, a diet rich in green vegetables, eggs, fish and omega-3-rich oils, accompanied by physical activity, is beneficial for patients with AMD and might slow disease progression and help maintain better visual functions. Further studies of larger cohorts might elucidate the potential effects of various other food components and nutritional factors on the severity and progression of AMD or even their roles in preventing this disease.

## Figures and Tables

**Table 1:** Characteristics of the study groups. In bold, *p*-value < 0.05, which was considered statistically significant.

Parameter	AMD Group	Control Group	*p*-Value *
**Number of patients**	330	121	-
**Sex (male/female)**	135/219	32/89	**0.02**
**Age [years] (mean ± SD)**	73.4 ± 8.0	73.1 ± 6.0	0.41
**Current smokers (%)**	13.6%	6.3%	0.05
**Former smokers (%)**	51.4%	30.9%	**<0.001**
**Smoking pack-years (mean ± SD)**	13.6 ± 18.9	6.0 ± 13.1	**<0.001**
**Period without smoking [years] (mean ± SD)**	6.8 ± 10.9	5.3 ± 10.2	0.06
**Iris colour (dark/light)**	91/261	26/95	0.39
**Outdoor/indor working conditions**	40.1/59.9%	33.1/66.9%	0.19
**MAP [mmHg] (mean ± SD)**	98.3 ± 11.1	98.7 ± 9.7	0.86
**Disease history:**			
**Hypertension (%)**	64.7%	71.1%	0.27
**Hypertension duration [years] (mean ± SD)**	8.2 ± 9.5	9.2 ± 9.9	0.27
**Ischemic heart disease (%)**	16.2%	11.3%	0.33
**Ischemic heart disease duration [years] (mean ± SD)**	1.2 ± 4.2	0.8 ± 3.3	0.26
**Aortic aneurysm (%)**	1.6%	0.0%	0.59
**Peripheral artery disease (%)**	5.0%	6.2%	0.61
**Cerebral stroke (%)**	2.8%	3.1%	1.00
**Cardiac infarction (%)**	6.2%	6.1%	1.00
**Currently taken medications:**			
**Hypotensive drugs/vasodilators**	65.0%	70.1%	0.39
**Cardiac/antiarrhythmic drugs**	13.9%	14.4%	0.87
**NSAIDs**	20.2%	19.6%	1.00
**Hormonal drugs**	17.1%	20.6%	0.45
**Thyroxine**	13.7%	20.6%	0.11
**Steroids**	1.9%	1.0%	1.00
**Other hormonal drugs**	1.3%	0.0%	0.58
**Statins**	26.6%	36.1%	0.07
**Antidepressants**	4.7%	5.2%	0.79
**Antiasthmatic drugs**	7.4%	3.1%	0.16
**BMI [kg/m^2^] (mean ± SD)**	26.9 ± 4.2	26.6 ± 3.7	0.43
**WHR [cm/cm] (mean ± SD)**	0.90 ± 0.10	0.88 ± 0.10	0.13
**Waist circumference [cm] (mean ± SD)**	103.3 ± 9.1	102.1 ± 7.3	0.33
**Intensive physical activity (MET)**	269.2 ± 824.2	173.5 ± 451.7	0.59
**Average physical activity (MET)**	490.3 ± 1151.9	433.8 ± 706.2	0.35
**Walking (MET)**	778.8 ± 914.3	785.6 ± 852.4	0.63
**Total physical activity (MET)**	1536.2 ± 2025.1	1382.5 ± 1493.5	0.97
**Cholesterol [mg/dL] (mean ± SD)**	204.4 ± 44.6	202.8 ± 43.3	0.99
**HDL[mg/dL] (mean ± SD)**	60.1 ± 14.0	59.8 ± 13.6	0.90
**LDL[mg/dL] (mean ± SD)**	119.9 ± 39.2	117.5 ± 37.5	0.71
**Triglycerides [mg/dL] (mean ± SD)**	106.2 ± 51.5	108.9 ± 52.4	0.38
**Glucose [mg/dL] (mean ± SD)**	104.8 ± 12.7	102.8 ± 11.0	0.12

* Mann—Whitney test/Fisher’s exact test.

**Table 2:** Dietary habits of the AMD group and control group. *p*-values < 0.05, which were considered statistically significant, are shown in bold.

Food Group	Portions Per Week (%)	AMD Group	Control Group	*p*-Value *
**Fatty fish**	<1	73.67%	59.57%	**0.008**
	2–4	26.02%	39.36%	
	>4	0.31%	1.06%	
**Eggs**	<1	23.82%	10.75%	**0.04**
	2–4	69.91%	84.95%	
	>4	6.27%	4.30%	
**Green vegetables**	<2	5.63%	1.08%	0.40
	2–7	63.44%	76.34%	
	>7	30.94%	22.58%	
**Fruit and fruit juice**	<2	6.94%	1.06%	**0.01**
	2–7	46.69%	72.34%	
	>7	46.37%	26.60%	
**Omega-3 rich oils**	<2	14.38%	4.21%	0.66
	2–7	64.38%	81.05%	
	>7	21.25%	14.74%	
**Simple carbohydrates**	<2	25.86%	11.58%	0.18
	2–7	55.45%	74.74%	
	>7	18.69%	13.68%	
**Complex carbohydrates**	<2	2.18%	3.16%	0.26
	2–7	77.26%	81.05%	
	>7	20.56%	15.79%	
**Beer consumption**	0	37.54%	36.08%	0.87
	≤1	58.68%	63.92%	
	2–7	3.79%	0.00%	
**Wine consumption**	0	37.78%	36.08%	0.88
	<2	60.95%	63.92%	
	2–7	1.27%	0.00%	
**Vodka consumption**	0	37.42%	36.08%	0.84
	≤1	62.26%	63.92%	
	2–3	0.31%	0.00%	

* Mann–Whitney test.

**Table 3:** Dietary habits of the early, intermediate and late AMD groups. The bold font indicates *p*-values < 0.05, which were considered statistically significant.

Food Group	*p*-Value ^1^	Portions Per Week (%)	Early AMD Group	Intermediate AMD Group	Late AMD Group	*p*-Value ^2^		
						Early AMD vs. Intermediate AMD	Early AMD vs. Late AMD	Intermediate AMD vs. Late AMD
**Fatty fish**	0.047	<1	76.47%	63.44%	77.46%	0.21	0.83	0.01
		2–4	20.59%	36.56%	22.54%			
		>4	2.94%	0.00%	0.00%			
**Eggs**	0.28	<1	11.76%	17.20%	28.90%	0.84	0.26	0.18
		2–4	88.24%	78.49%	63.01%			
		>4	0.00%	4.30%	8.09%			
**Green vegetables**	0.47	<2	0.00%	3.23%	7.47%	0.45	0.24	0.55
		2–7	64.71%	66.67%	63.22%			
		>7	35.29%	30.11%	29.31%			
**Fruit and fruit juice**	0.83	<2	2.94%	6.52%	8.72%	0.53	0.67	0.76
		2–7	50.00%	51.09%	45.35%			
		>7	47.06%	42.39%	45.93%			
**Omega-3 rich oils**	0.25	<2	5.88%	8.60%	18.39%	0.14	0.12	0.66
		2–7	64.71%	74.19%	58.62%			
		>7	29.41%	17.20%	22.99%			
**Simple carbohydrates**	0.36	<2	8.82%	22.34%	30.46%	0.29	0.17	0.59
		2–7	76.47%	61.70%	49.43%			
		>7	14.71%	15.96%	20.11%			
**Complex carbohydrates**	0.48	<2	0.00%	0.00%	4.02%	0.18	0.40	0.57
		2–7	73.53%	84.04%	73.56%			
		>7	26.47%	15.96%	22.41%			
**Beer consumption**	0.75	0	42.86%	36.17%	38.46%	0.46	0.64	0.64
		≤1	54.29%	59.57%	58.58%			
		2–7	2.86%	4.26%	2.96%			
**Wine consumption**	0.81	0	42.86%	36.56%	38.69%	0.56	0.76	0.63
		<2	54.29%	61.29%	60.71%			
		2–7	2.86%	2.15%	0.60%			
**Vodka consumption**	0.74	0	42.86%	36.17%	38.24%	0.45	0.61	0.66
		≤1	57.14%	62.77%	61.76%			
		2–3	0.00%	1.06%	0.00%			

^1^ Kruskal-Wallis test, ^2^ Mann-Whitney test.

**Table 4:** Comparison of levels of components of the antioxidant system in patients with AMD and controls. The bold font indicates *p*-values < 0.05, which were considered statistically significant.

Antioxidant System Component	AMD Group	Control Group	*p*-Value *
	Mean ± SD	Mean ± SD	
**SOD (RBC) [U/mg Hb]**	0.31 ± 0.23	0.33 ± 0.19	0.56
Catalase (RBC) [U/mg Hb]	0.33 ± 0.21	0.53 ± 0.24	**<0.0001**
**GPx (RBC) [U/g Hb]**	0.05 ± 0.05	0.02 ± 0.01	**<0.0001**
**R-GSSG (RBC) [U/g Hb]**	7.28 ± 5.73	5.55 ± 2.62	**0.001**
GSH (RBC) [µmol/g Hb]	6.18 ± 2.7	6.49 ± 1.95	0.13
GSH transferase (RBC) [U/g Hb]	0.02 ± 0.02	0.01 ± 0.01	**<0.0001**
SOD (PLT) [U/mg of protein]	69.24 ± 145.07	29.07 ± 26.64	**0.03**
Catalase (PLT) [U/mg of protein]	28.31 ± 72.11	9.08 ± 17.91	**<0.0001**
GPx (PLT) [U/g of protein]	4.26 ± 16.083	1.45 ± 1.99	**0.002**
R-GSSG (PLT) [U/g of protein]	1604.07 ± 5624.53	515.63 ± 528.46	**0.04**
GSH (PLT) [µmol/g Hb]	1964.62 ± 3398.87	1126.1 ± 2141.95	0.44
GSH transferase (PLT) [U/g of protein]	1.83 ± 3.92	1.06 ± 1.12	0.26

* Mann-Whitney test.

**Table 5:** Comparison of levels of components of the antioxidant system in patients with early, intermediate and late AMD. *p*-values < 0.05, which were considered statistically significant, are shown in bold.

Antioxidant System Component	*p*-Value ^1^	Early AMD Group		Intermediate AMD Group		Late AMD Group		*p*-Value ^2^		
		*n*	Mean ± SD Median (IQR)	*n*	Mean ± SD Median (IQR)	*n*	Mean ± SD Median (IQR)	Early AMD vs. Intermediate AMD	Early AMD vs. Late AMD	Intermediate AMD vs. Late AMD
**SOD (RBC) [U/mg Hb]**	0.27	39	0.27 ± 0.25	99	0.33 ± 0.23	184	0.30 ± 0.23	0.11	0.26	0.32
			0.15 (0.38)		0.33 (0.43)		0.26 (0.42)			
**Catalase (RBC) [U/mg Hb]**	0.12	39	0.38 ± 0.25	99	0.36 ± 0.22	184	0.32 ± 0.20	0.83	0.15	0.07
			0.31 (0.31)		0.30 (0.31)		0.26 (0.24)			
**GPx (RBC) [U/g Hb]**	0.17	39	0.04 ± 0.04	99	0.04 ± 0.04	183	0.05 ± 0.06	0.46	0.09	0.22
			0.02 (0.01)		0.02 (0.02)		0.02 (0.07)			
**R-GSSG (RBC) [U/g Hb]**	0.97	39	7.27 ± 4.54	99	6.59 ± 2.86	185	7.64 ± 7.12	0.94	0.82	0.86
			6.18 (5.64)		6.20 (3.23)		6.07 (5.07)			
**GSH (RBC) [µmol/g Hb]**	0.08	39	6.99 ± 2.38	99	6.56 ± 3.49	185	5.93 ± 2.25	0.09	**0.03**	0.47
			6.89 (2.94)		6.26 (3.59)		6.36 (3.96)			
**GSH transferase (RBC) [U/g Hb]**	0.38	39	0.02 ± 0.02	99	0.02 ± 0.02	185	0.02 ± 0.02	0.26	0.16	0.82
					0.01 (0.02)		0.01 (0.02)			
			0.01 (0.01)							
**SOD (PLT) [U/mg of protein]**	0.31	39	44.82 ± 55.41	98	57.93 ± 85.41	186	84.88 ± 184.59	0.48	0.14	0.40
			24.35 (39.32)		26.83 (43.76)		27.49 (76.11)			
**Catalase (PLT) [U/mg of protein]**	0.13	39	14.81 ± 19.31	98	21.68 ± 35.18	186	35.54 ± 93.80	0.14	0.05	0.51
			6.37 (16.70)		9.62 (13.72)		9.95 (31.73)			
**GPx (PLT) [U/g of protein]**	0.37	38	1.71 ± 1.77	98	3.98 ± 14.11	185	5.27 ± 19.33	0.80	0.32	0.23
			1.28 (2.00)		0.94 (2.94)		1.20 (2.66)			
**R-GSSG (PLT) [U/g of protein]**	0.12	39	1478.05 ± 5657.85	97	1914.16 ± 8866.18	185	1499.27 ± 3268.86	0.15	**0.04**	0.52
			281.19 (750.52)		472.54 (785.88)		466.47 (1022.51)			

*n*: number of observations, ^1^ Kruskal-Wallis test, ^2^ Mann-Whitney test.

## Abbreviations

**AMD**	age-related macular degeneration
**CAT**	catalase
**CNV**	choroidal neovascularization
**GA**	geographic atrophy
**GPx**	glutathione peroxidase
**R-GSSG**	glutathione reductase
**GST**	glutathione transferase
**nAMD**	neovascular AMD
**PLT**	platelets
**ROS**	reactive oxygen species
**RBCs**	red blood cells
**GSH**	reduced glutathione
**RPE**	retinal pigment epithelium
**SOD**	superoxide dismutase
